# Prevalence and Predictors of Overweight and Obesity among Somalis in Norway and Somaliland: A Comparative Study

**DOI:** 10.1155/2018/4539171

**Published:** 2018-09-03

**Authors:** Soheir H. Ahmed, Haakon E. Meyer, Marte K. Kjøllesdal, Ahmed A. Madar

**Affiliations:** ^1^Department of Community Medicine and Global Health, Institute of Health and Society, University of Oslo, Oslo, Norway; ^2^College of Medicine & Health Science, University of Hargeisa, Hargeisa, Somaliland; ^3^Division of Mental and Physical Health, Norwegian Institute of Public Health, Oslo, Norway

## Abstract

**Background and Aim:**

The knowledge about the health status of Somalis in Norway and Somaliland is limited. This paper reports the results of a comparative study on the prevalence and predictors of overweight/obesity among Somalis in Norway and Somaliland.

**Method:**

We conducted two cross-sectional studies using the same tools and procedures, between 2015 and 2016. The study population was adults aged 20–69 years (*n*=1110 (Somaliland) and *n*=220 (Norway)).

**Results:**

The prevalence of obesity (body mass index (BMI) ≥30 kg/m^2^) was 44% and 31% in women in Norway and Somaliland, respectively. In contrast, the prevalence of obesity was low in men (9% in Norway; 6% in Somaliland). Although the prevalence of high BMI was higher in Somali women in Norway than women in Somaliland, both groups had the same prevalence of central obesity (waist circumference (WC) ≥ 88 cm). In men, the prevalence of central obesity (WC ≥ 102 cm) was lower in Somaliland than in Norway. For women in Somaliland, high BMI was associated with lower educational level and being married.

**Conclusion:**

The prevalence of overweight and obesity is high among Somali immigrants in Norway, but also among women in Somaliland. The high prevalence of overweight and obesity, particularly among women, calls for long-term prevention strategies.

## 1. Introduction

Overweight and obesity are regarded as serious threats to public health which significantly increase the risk of noncommunicable diseases (NCDs) such as cardiovascular disease (CVD), type-2 diabetes (T2D), hypertension, and certain cancers [1]. The World Health Organization (WHO) estimates that overweight and obesity are the fifth leading cause of death globally. Today, nearly two billion adults worldwide are overweight or obese [2]. Along with increased body mass index, waist circumference (WC) and waist-hip ratio (WHR) are accepted as alternative predictive measurements of NCD [3].

Overweight and obesity are not only a problem in developed countries but are also dramatically on the rise in low- and middle-income countries, particularly in urban settings [4]. A high prevalence of overweight and obesity has been identified in sub-Saharan Africa (SSA), especially among women and urban dwellers [5]. Currently, there are no official data about the prevalence of overweight and obesity among the Somali population in the Horn of Africa. However, according to WHO estimates, the prevalence is not high [6].

In Norway, studies have demonstrated that non-Western immigrants tend to adopt the negative aspects of a Western lifestyle, including poor eating habits and a sedentary lifestyle, and thus become at high risk of overweight and obesity [7–9]. In women, the prevalence of overweight and obesity among immigrants is high compared to ethnic Norwegians; however, there are large differences between immigrant groups [10]. Somalis are one of the largest non-Western immigrant groups in Norway, with most of them migrating due to their country's civil war, which started in the late 1980s [11]. Therefore, they are a relatively new immigrant group, and the knowledge about their health status in both the host country and their country of origin is limited. Results from a few studies have shown that many Somali immigrants are overweight and obese due to nutritional transition, lack of physical activity, and other factors [12, 13].

To our knowledge, no studies have yet compared the prevalence of overweight and obesity among Somali immigrants with their counterparts in the country of origin. The aim of this article was to compare the prevalence and predictors of overweight and obesity among Somalis in Oslo, Norway, and Somalis in Hargeisa, Somaliland.

## 2. Methods

This is a comparative cross-sectional study conducted between December 2015 and October 2016 in Norway and between March and September 2016 in Somaliland. In both studies, participants were excluded if they confirmed to be pregnant or were suffering from kidney or liver failure, cancer, and other serious diseases.

### 2.1. Study Population and Recruitment

#### 2.1.1. Study 1, Oslo, Norway

The majority of Somali immigrants live mainly in eastern and central districts of Oslo. Experience from other studies on immigrant populations has shown that drawing a random sample from the Statistics Norway (SSB) and contacting possible participants through written information does not work well in many immigrant groups. Therefore, for organizational purposes, this study was limited to Sagene district, which has one of the highest populations of Somali origin in the city. Cooperation with Somali organizations, a healthy life centre, a volunteer centre, the district medical officer, and the community development centre in Sagene area was established. These user partners contributed to the recruitment of participants in this study. Information of the study was shared through local Somali radio, community centres in the district, and other locations. We could not get the exact numbers of Somalis in the district because people move frequently without reporting their new address, and the statistics data at the district level are not updated often. However, in 2015, there were 1200 persons with Somali background in all ages registered in the district. An attempt to contact every adult person of Somali background living in the district was made, and those available were invited to participate in the study. We ended up including 221 persons, and 50 persons either did not want to participate or did not come for the appointment. The participants were healthy adult men and women of Somali origin, aged 20–69 years. The response rate of the participants was 82% (221/271).

#### 2.1.2. Study 2, Hargeisa, Somaliland

There is no population registry in Somaliland, and the only available registry is the number of households. Each household has a unique number, and the number of people residing in the household is registered at the district level. Hargeisa city composes of five major districts, of which each district is further subdivided into four main subdistricts. These subdistricts are the primary sampling unit (PSU). Due to the lack of data on the prevalence of the risk factors on the population under study, the sample size was calculated using the diabetes prevalence of 4%. The sample design for the survey was two-stage cluster sampling. The first stage units were subdistricts designated as the PSUs, and the second stage units were households. The number of PSUs targeted was twenty subdistricts, and the number of all households in the targeted PSUs was 66635 (households in all subdistricts). Out of the twenty PSUs, ten were randomly selected. A total of 1100 households were randomly selected from the ten subdistricts based on the probability proportionate to size (PPS) in each subdistrict [14]. In each household selected, all the eligible persons (20–69 years) living in the house were listed in a Kish household coversheet. The Kish method addresses the selection of gender and different age-groups in the sample. Men and women were listed in order of decreasing age (oldest to youngest) and given a rank number. Then, the Kish selection table was applied to select the eligible participant whose rank number matched with the last digit of the household. If the selected person rejected participation, another person was selected from the Kish list and continued until one person from each household was included in the study. If there was nobody at home on the day of the study, a notification card was left at the door, and we returned the next day until we had a participant from each house. Data collection continued until there were 1100 participants, resulting in a final sample of 955 women and 145 men.

### 2.2. Data Collection

The two studies followed similar data collection methods and used the same tools. The Hargeisa study followed the WHO STEPwise approach to chronic disease risk factor surveillance [14]. Data collection was conducted by researchers and trained fieldworkers. Participants from Oslo and Hargeisa were interviewed using a structured questionnaire. Age, education, occupation, and marital status were reported.

In both studies, body weight was measured to the nearest 0.1 kg by an electronic Omron medical scale, height was measured to the nearest 0.5 cm with participants standing without shoes using a portable stadiometer seca 213, and body mass index (BMI) was calculated as weight in kilograms divided by the square of the height in meters (kg/m^2^).

WC was measured at the midpoint between the lower margin of the last palpable rib and the top of the iliac crest, using a stretch to the nearest 0.1 cm with the subject standing and breathing normally. Hip circumference (HC) was measured around the widest portion of the buttocks with the tape parallel to the floor. WHR was calculated by dividing WC by HC.

BMI was categorized according to World Health Organization classification: underweight (<18.5 kg/m^2^), normal (BMI 18.5–24.9 kg/m^2^), overweight (BMI 25–29.9 kg/m^2^), and obese (BMI ≥ 30 kg/m^2^) [15]. Central obesity was defined as WC ≥ 88 cm for women and ≥102 cm for men as well as WHR ≥ 0.85 for women and ≥1.00 for men [16].

### 2.3. Ethics Statement

Both studies were approved by the Regional Committee for Medical and Health Research Ethics, Norway. In addition, the Somaliland study was approved by the Ministry of Health in Somaliland. Permission to conduct the study was obtained from the local government of the municipality and household level. In both studies, written informed consent was obtained from all participants.

### 2.4. Statistical Analysis

Combined data were analysed by IBM SPSS statistical software-24 (SPSS Inc., Chicago, Illinois, USA). Descriptive statistics are presented as mean (SD) and percentage. We compared categorical variables using *χ*^2^*test* and independent *t*-test for continuous variables. We compared age-adjusted means for anthropometric measurements using analysis of covariance (ANCOVA). The relationship between BMI and associated variables (location, gender, age, education, marital status, and occupation) were tested using linear regression model. In addition, age-adjusted prevalence ratio was calculated in STATA version 14 by generalized linear model (log-binomial regression) logarithmic link function. A *p* value of <0.05 was considered statistically significant.

## 3. Results

### 3.1. Background Characteristics

A total of 1320 respondents were included in the analysis, with 1100 from Hargeisa and 220 from Oslo.

In Table 1, sociodemographic characteristics of the study population are shown. In Hargeisa, more women than men were included, whereas the proportion of men and women included in Oslo was equal. The mean age (years) in men and women was similar among the groups. The educational level was relatively low among participants from Hargeisa, particularly among women where two-thirds had no formal education. Moreover, unemployment was high among women from Hargeisa (88.5%), as most of the participants were housewives. Around one-third of the men in Hargeisa and one-fifth of the men in Oslo were unemployed.

### 3.2. Anthropometric Characteristics and Prevalence of Obesity

**Figure 1:**
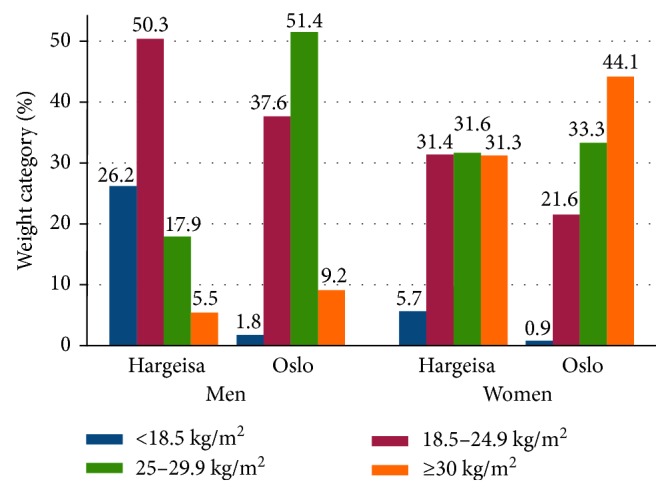
Crude prevalence of weight categories among Oslo and Hargeisa participants.

Somali men in Oslo were taller and had higher mean weight, BMI, WC, HC, and WHR compared to their counterparts in Hargeisa (Table 2). While the prevalence of obesity among men was higher in Oslo compared to Hargeisa (9.2% versus 5.5%), the prevalence of underweight was substantially higher among men in Hargeisa than in Oslo (26.2% versus 1.8%) (Figure 1). Mean BMI was considerably higher in women compared to men in both locations. Women in Oslo had higher weight, BMI, and HC than women in Hargeisa, but women had similar height and WC in both locations. WHR was higher in women from Hargeisa than in women from Oslo, whereas 44.1% of women in Oslo were obese, and the corresponding prevalence in Hargeisa was 31.3%. Central obesity measured by WC and WHR was higher among men in Oslo (31.8% and 12.7%) compared to men in Hargeisa (6.2% and 6.9%). Central obesity measured as WC was similar among women in Oslo and Hargeisa (50.9% and 49.5%). However, central obesity measured as WHR was higher among women in Hargeisa (44.2%) compared to women in Oslo (28.6%) (Table 2). Additional analyses adjusting for age gave similar results as those presented in Table 2 and Figure 1 (data not shown).

### 3.3. Predictors of BMI

In both genders, higher BMI was associated with living in Oslo and increasing age (Table 3). In women, higher BMI was also associated with being married, whereas lower BMI was associated with being a student. Examining BMI in different educational groups according to location and gender showed that among women in Hargeisa, mean BMI was higher among those with lower education (1.77 (0.24, 3.32)) and slightly higher among those with medium education (1.93 (−0.01, 3.88)) compared to women with high education (university) (Table 4). No associations with education were found in the other groups. Additional analysis showed that, BMI was higher among married women and housewives in Hargeisa when compared to women in Oslo and men in both groups (data not shown).

## 4. Discussion

Our study demonstrated a high prevalence of overweight and obesity among women in both populations, especially in Oslo where nearly one in two women were obese. The prevalence of obesity was considerably lower in men than women, especially among men in Hargeisa where one in four men was underweight. In addition, men in Hargeisa had a low prevalence of central obesity. However, this must be interpreted with caution, as the number of male participants in Hargeisa was low. Despite a higher BMI among women in Oslo than that in Hargeisa, the prevalence of central obesity measured by WC was the same between the two groups, and WHR was higher among women in Hargeisa than those in Oslo.

To our knowledge, this is the first study comparing the prevalence of overweight, obesity, and associated factors among Somali immigrants in Oslo and their counterparts in Hargeisa. Generally, the knowledge of the health status of Somalis in both the diaspora and the Horn of Africa is limited. The high prevalence of overweight and obesity among Somali immigrants in this study, especially among women, is in line with the few studies conducted among Somali immigrants in other Western countries [13, 17]. Although the prevalence of overweight and obesity was lower in Hargeisa compared to Oslo, the prevalence of overweight and obesity among women in Hargeisa was much higher than the estimates in Global Burden of Disease (GBD) data, which were obtained from the Somali Multiple Cluster Survey [18].

The higher prevalence of overweight and obesity among women was also reported in others studies in sub-Saharan Africa (SSA) [19, 20]. Also in other ethnic immigrant groups from developing countries living in Norway, a higher prevalence of obesity has been reported among women than among men [10]. In contrast, the prevalence of obesity among ethnic Norwegians is slightly higher in men than in women [21]. The prevalence of obesity in ethnic Norwegian men was somewhat higher than in Somali men in Oslo, whereas the prevalence of obesity was much higher among Somali women in Oslo than among ethnic Norwegian women [22]. Furthermore, Somali men in Oslo had higher prevalence of overweight (51.4%) compared to men in Hargeisa (17.9%), which may be indicative of an increase in obesity in the future if no prevention measurements are taken.

It has been reported that immigrants from SSA are at increased risk of overweight and obesity-related diseases after immigration to industrialised countries [23, 24]. In Norway, studies have demonstrated that non-Western immigrants tend to adopt the negative aspects of a Western lifestyle, including poorer eating habits and an increased sedentary lifestyle [7–9]. The underlying causes of the high prevalence of obesity among Somali women in Oslo can be multifactorial, including an adoption of a sedentary lifestyle and the rapid “acculturation” of poor dietary habits, characterized by foods of low nutritional quality, high caloric density, and high saturated fat. Nevertheless, little is known about Somali diet and cultural behaviours both in Norway and Somaliland. Traditionally, Somali food consists of pasta, rice, and red meat. Additionally, tea with large quantity of sugar is an essential drink in their daily life [25]. The high prevalence of obesity among women in Hargeisa can also be due to urbanization and a rapid nutrition transition.

In the present study, Somali women in Oslo had a lower WHR compared to women in Hargeisa. However, WC was similar, and the difference in WHR was driven by a higher HC among women in Oslo. In other words, despite a higher BMI, women in Oslo had more mass accumulated on the hips than on the waist compared to women in Hargeisa. Their risk of future diseases like diabetes and cardiovascular disease might therefore be lower than anticipated from BMI alone. Some studies have recommended that proper cutoff points for BMI and anthropometric measures may need to be established for SSA populations [26, 27].

The relationship between BMI and education is nonlinear [28]. In Hargeisa, women with lower and middle education had higher mean BMI compared to those with higher education. However, both for men in Oslo and Hargeisa and for women in Oslo, results did not differ significantly. These results are contrary to the findings in other SSA countries, where BMI has been associated with higher educational level [29, 30], while other study found that women with no education and higher education had lower BMI when compared to those with some schooling [31]. One explanation for our findings is that less educated women were married housewives and might eat more calorie-dense food or have inactive lifestyle than more highly educated women. However, there was no association between mean BMI and other socioeconomic status (SES) among other participants. Further studies are needed to investigate the relationship between BMI and SES in this population.

### 4.1. Strengths and Weaknesses

Our study has several strengths. Similar design and standardised tools were used in the two studies, facilitating the comparisons of groups in different settings. The same project leaders in Oslo and Hargeisa conducted and supervised the teams during data collection. The weight scales were checked every morning. The Hargeisa study is the first population-based study that has been carried out in Somaliland that used the WHO STEPwise approach to noncommunicable disease risk factor surveillance (STEPS) [14].

A limitation of our study was the underrepresentation of men in Hargeisa. They constituted only 15% of the sample. Most of the men were away from home at the time of the study. If the selected men were not home, we left a notification letter that the team would come back the next day. But if they were not present the next day, we selected the next eligible person from the Kish list. Moreover, 50 eligible men refused to be included in the study. According to Somali culture, women are in the houses during the daytime and men are away working or socializing with other men. The men included might therefore have been men who were home for a special reason, such as poor health, and therefore, a selection bias may be present.

The Hargeisa study was only carried out in an urban setting. The sample was drawn from a big city with inhabitants from all Somali regions. Thus, we believe that our results may possibly be representative for cities all over Somaliland. Formerly, before separation between Somaliland and Somalia, Hargeisa was the second biggest city in Somalia. Regardless of political differences, the findings could also possibly be applied for regions in today's Somalia as Somalis are homogeneous groups, sharing social behaviour associated with overweight and obesity such as culture, tradition, language, religion, food habits, and other attitudes.

In the Oslo study, an attempt to contact every adult person with Somali background living in the district was made. Although the Norwegian Population Registry is of high quality in many aspects, the living address is often not up to date as immigrants frequently move within the city or within the country. On the other hand, comparison of the education levels and age distribution of included participants to data from Statistics Norway suggest that the participants included in the present study seem to be representative of adults with Somali background living in Norway.

## 5. Conclusion

The prevalence of overweight and obesity was high among Somali immigrants in Oslo but also among women in Hargeisa. The high prevalence of overweight and obesity, particularly among women, calls for long-term prevention strategies. Achieving reductions in overweight and obesity rates for Somali people who are in the midst of a nutrition transition and who are immigrants is of critical importance in lowering high obesity-related social and healthcare costs, as well as morbidity and mortality. The sociodemographic factors associated with overweight and obesity in Somali population requires further investigation.

## Figures and Tables

**Table 1:** Sociodemographic characteristics of the study population in Oslo and Hargeisa.

Men, *N*	Oslo, 109	Hargeisa, 145	*p* value
Age, mean (SD)	39.7 (11.7)	38.2 (16.1)	0.409
Education, (%)			
No education	0	31.7	<0.01
Primary	10.9	15.9	
Secondary	52.7	21.4	
University	36.3	31	
Occuption (%)			
Unemployed	19.1	32.2	<0.01
Employed	61.8	41.3	
Students	10.9	12.6	
Others (sick/maternal leave and retired)	8.1	14	
Marital status (%)			
Not married	27.3	41.4	0.02
Married	65.5	56.5	
Others (separate and divorced)	7.3	2.1	

Women, *N*	Oslo, 111	Hargeisa, 955

Age, mean (SD)	37.7 (10.1)	39.1 (14.1)	0.312
Education (%)			
No education	2.7	66.4	<0.01
Primary	50.0	19.4	
Secondary	30.4	7.7	
University	17	6.5	
Occupation (%)			
Unemployed	22.7	88.5	0.01
Employed	24.5	6.0	
Students	36.4	4.1	
Others (sick/maternal leave and retired)	16.3	1.5	
Marital status (%)			
Not married	25.0	17.3	0.01
Married	64.3	78.4	
Others (separated and divorced)	10.7	4.3	

*χ* ^2^ test for categorical variables and *t*-test for continuous variables.

**Table 2:** Crude anthropometric characteristics, obesity prevalence (%), and central obesity prevalence (%) for Oslo and Hargeisa.

Men	Oslo (*n*=109)	Hargeisa (*n*=145)	*p* value^*∗*^
Weight (cm), mean (SD)	79.9 (11.1)	65.0 (13.2)	<0.001
Height (cm), mean (SD)	175.5 (6.7)	172.9 (7.5)	0.005
Body mass index (kg/m^2^)			
Mean (SD)	25.9 (3.3)	22.1 (4.5)	<0.001
Prevalence of obesity (≥30 kg/m^2^), %	9.2	5.5	
Waist circumference (cm)			
Mean (SD)	95.5 (10.5)	81.9 (13.6)	<0.001
Central obesity (≥102 cm), %	31.8	6.2	
Hip circumference (cm), mean (SD)	104.5 (7.4)	93.9 (11.2)	<0.001
Waist-hip ratio			
Mean (SD)	0.91 (0.07)	0.87 (0.09)	<0.001
Central obesity (≥1.00), %	12.7	6.9	

Women	Oslo (*n*=111)	Hargeisa (*n*=955)	

Weight (cm), mean (SD)	77.4 (16.3)	70.7 (15.9)	<0.001
Height (cm), mean (SD)	161.1 (6.7)	161.0 (6.0)	0.954
Body mass index (kg/m^2^),			
Mean (SD)	30.0 (6.8)	27.2 (5.9)	<0.001
Prevalence of obesity (≥30 kg/m^2^), %	44.1	31.3	
Waist circumference (cm)			
Mean (SD)	88.6 (15.3)	87.1 (14.3)	0.293
Central obesity (≥88 cm), %	50.9	49.5	
Hip circumference (cm), mean (SD)	110.0 (13.3)	103.7 (12.6)	<0.001
Waist-hip ratio			
Mean (SD)	0.80 (0.09)	0.84 (0.09)	<0.001
Central obesity (≥0.85), %	28.6	44.2	

^*∗*^ *p* value for the comparison of means.

**Table 3:** Associations between body mass index (BMI) and sociodemographic factors from linear regressions.

Covariates	Men (*n*=254)	Women (1066)
	*β*-Coefficient (95% CI)	*β*-Coefficient (95% CI)
Age	0.09 (0.05, 0.13)^*∗*^	0.09 (0.06, 0.11)^*∗*^
Location^a^ (ref: Hargeisa)		
Oslo	3.75 (2.77, 4.72)^*∗*^	2.86 (1.71, 4.01)^*∗*^
Education^b^ (university)		
No/preschool	−0.66 (−1.99, 68)	1.65 (0.26, 3.04))^*∗∗*^
Secondary	−0.73 (−1.91, 0.46)	1.48 (−0.22, 3.18)
Marital status^b^ (ref: single)		
Married	0.92 (−0.36, 2.19)	2.88 (1.90, 3.86)^*∗∗*^
Divorced/widowed	−1.20 (−3.83, 1.43)	0.95 (−0.82, 2.72)
Occupation^b^ (ref: unemployed)		
Employed	−0.22 (−1.59, 0.69)	0.50 (−1.89, 1.16)
Students	−2.14 (−3.91, 0.37)	−2.56 (−4.09, −1.03)
Retired	−0.59 (−2.68, 1.50)	−0.19 (−2.39, 2.01)

^a^Location adjusted for age. ^b^Adjusted for location and age. ^*∗*^*p*  value < 0.001.  ^*∗∗*^*p*  value < 0.05.

**Table 4:** Age-adjusted mean BMI (95% CI) and prevalence ratio (PR) (95% CI) with education.

	*N*	Mean BMI (95% CI)	Difference (95% CI) BMI within education levels	PR	(95% CI)
*Men*					
Oslo	109				
Education					
Preschool/primary^*∗*^	12	26.3 (24.4, 28.2)	0.18 (−1.97, 2.34)	1.02	0.12, 8.86
Secondary	57	25.6 (24.9, 26.6)	−0.42 (−1.77, 0.93)	1.33	0.35, 5.01
University	40	26.1 (25.1, 27.2)	1.00 (ref)	1.00 (ref)	1.00 (ref)
Hargeisa	145				
Education					
Preschool/primary	69	21.6 ((20.5, 22.7)	−1.23 (−3.05, 0.60)	0.68	0.13 ,3.58
Secondary	31	21.9 (20.3, 23.4)	−0.97 (−2.96, 1.02)	0.50	0.05, 4.50
University	45	22.9 (21.5, 24.2)	1.00 (ref)	1.00 (ref)	1.00 (ref)
*Women*					
Oslo	111				
Education					
Preschool/primary	59	30.6 (28.9, 32.3)	1.42 (−2.06, 4.90)	1.60	0.72, 3.56
Secondary	33	29.4 (27.1, 31.6)	0.17 (−3.63, 3.97)	1.99	0.89, 4.46
University	19	29.2 (26.1, 32.2)	1.00 (ref)	1.00 (ref)	1.00 (ref)
Hargeisa	955				
Education					
Preschool/primary	819	27.2 (26.9, 27.7)	1.77 (0.24, 3.32)	1.59	0.89, 2.84
Secondary	74	27.4 (26.2, 28.8)	1.93 (−0.01, 3.88)	1.83	0.96, 3.52
University	62	25.5 (24.1, 27.0)	1.00 (ref)	1.00 (ref)	1.00 (ref)

^*∗*^No schooling and primary education.

## Abbreviations

BMI: Body mass index
CVD: Cardiovascular diseases
NCD: Noncommunicable diseases
PSUs: Primary sample units
PPS: Probability proportionate to size
T2D: Type-2 diabetes
WC: Waist circumference
WHO: World Health Organization
WHR: Waist-hip ratio.

## References

[1] Guh D. P., Zhang W., Bansback N., Amarsi Z., Birmingham C. L., Anis A. H. (2009). The incidence of co-morbidities related to obesity and overweight: a systematic review and meta-analysis. *BMC Public Health*.

[2] WHO (2017). Obesity and Overweight. http://www.who.int/mediacentre/factsheets/fs311/en/.

[3] Czernichow S., Kengne A. P., Stamatakis E., Hamer M., Batty G. D. (2011). Body mass index, waist circumference and waist-hip ratio: which is the better discriminator of cardiovascular disease mortality risk?: evidence from an individual-participant meta-analysis of 82 864 participants from nine cohort studies. *Obesity Reviews*.

[4] Adeboye B., Bermano G., Rolland C. (2012). Obesity and its health impact in Africa: a systematic review. *Cardiovascular Journal Of Africa*.

[5] Scott A., Ejikeme C. S., Clottey E. N., Thomas J. G. (2013). Obesity in sub-Saharan Africa: development of an ecological theoretical framework. *Health Promotion International*.

[6] WHO (2014). *Noncommunicable Diseases Country Profiles*.

[7] Holmboe-Ottesen G., Wandel M. (2012). Changes in dietary habits after migration and consequences for health: a focus on South Asians in Europe. *Food & Nutrition Research*.

[8] Råberg M., Kumar B., Holmboe-Ottesen G., Wandel M. (2009). Overweight and weight dissatisfaction related to socio-economic position, integration and dietary indicators among South Asian immigrants in Oslo. *Public Health Nutrition*.

[9] Jenum A. K., Holme I., Graff-Iversen S., Birkeland K. I. (2005). Ethnicity and sex are strong determinants of diabetes in an urban Western society: implications for prevention. *Diabetologia*.

[10] Kumar B. N., Meyer H. E., Wandel M., Dalen I., Holmboe-Ottesen G. (2006). Ethnic differences in obesity among immigrants from developing countries, in Oslo, Norway. *International Journal of Obesity*.

[11] SSB (2017). *Immigrants and Norwegian-Born to Immigrant Parents*.

[12] Torp J. A., Berggren V., Erlandsson L.-K., Westergren A. (2015). Weight status among Somali immigrants in Sweden in relation to sociodemographic characteristics, dietary habits and physical activity. *The Open Public Health Journal*.

[13] Guerin P. B., Elmi F. H., Corrigan C. (2007). Body composition and cardiorespiratory fitness among refugee Somali women living in New Zealand. *Journal of Immigrant and Minority Health*.

[14] WHO (2017). *The WHO Stepwise Approach to Noncommunicable Disease Risk Factor Surveillance*.

[15] WHO (2017). *The International Classification of Adult Underweight, Overweight and Obesity According to BMI*.

[16] WHO (2008). *Waist Circumference and Waist–Hip Ratio*.

[17] Gele A. A., Mbalilaki A. J. (2013). Overweight and obesity among African immigrants in Oslo. *BMC Research Notes*.

[18] Healthdata.org (2015). Prevalence of overweight and obesity-Somalia. https://vizhub.healthdata.org/obesity/.

[19] Kirunda B. E., Fadnes L. T., Wamani H., Van den Broeck J., Tylleskär T. (2015). Population-based survey of overweight and obesity and the associated factors in peri-urban and rural Eastern Uganda. *BMC Public Health*.

[20] Abubakari A. R., Lauder W., Jones M. C., Kirk A., Agyemang C., Bhopal R. S. (2008). Prevalence and time trends in obesity among adult West African populations: a meta-analysis. *Obes Rev*.

[21] Kumar B. N., Selmer R., Lindman A. S., Tverdal A., Falster K., Meyer H. E. (2009). Ethnic differences in SCORE cardiovascular risk in Oslo, Norway. *European Journal of Cardiovascular Prevention & Rehabilitation*.

[22] Meyer H. E., Tverdal A. (2005). Development of body weight in the Norwegian population. *Prostaglandins, Leukotrienes and Essential Fatty Acids*.

[23] Saleh A., Amanatidis S., Samman S. (2002). The effect of migration on dietary intake, type 2 diabetes and obesity: the Ghanaian health and nutrition analysis in Sydney, Australia (Ghanaisa). *Ecology of Food and Nutrition*.

[24] Agyemang C., Meeks K., Beune E. (2016). Obesity and type 2 diabetes in sub-Saharan Africans–is the burden in today’s Africa similar to African migrants in Europe? The RODAM study. *BMC Medicine*.

[25] McEwen A., Straus L., Croker H. (2009). Dietary beliefs and behaviour of a UK Somali population. *Journal of Human Nutrition and Dietetics*.

[26] Kamadjeu R. M., Edwards R., Atanga J. S., Kiawi E. C., Unwin N., Mbanya J. C. (2006). Anthropometry measures and prevalence of obesity in the urban adult population of Cameroon: an update from the Cameroon Burden of Diabetes Baseline Survey. *BMC Public Health*.

[27] Puoane T., Steyn K., Bradshaw D. (2002). Obesity in South Africa: the South African Demographic and Health Survey. *Obesity Research*.

[28] Stunkard A. J., Sørensen T. I. (1993). Obesity and socioeconomic status–a complex relation. *New England Journal of Medicine*.

[29] Neupane S., Prakash K. C., Doku D. T. (2016). Overweight and obesity among women: analysis of demographic and health survey data from 32 Sub-Saharan African countries. *BMC Public Health*.

[30] Sartorius B., Veerman L. J., Manyema M., Chola L., Hofman K. (2015). Determinants of obesity and associated population attributability, South Africa: Empirical Evidence from a National Panel Survey, 2008–2012. *PLoS One*.

[31] Micklesfield L. K., Lambert E. V., Hume D. J. (2013). Socio-cultural, environmental and behavioural determinants of obesity in black South African women. *Cardiovascular Journal of Africa*.

